# Retraction Note: Farnesyl dimethyl chromanol targets colon cancer stem cells and prevents colorectal cancer metastasis

**DOI:** 10.1038/s41598-025-27189-1

**Published:** 2025-11-10

**Authors:** Kazim Husain, Domenico Coppola, Chung S. Yang, Mokenge P. Malafa

**Affiliations:** 1 Department of Gastrointestinal Oncology, H. Lee Moffitt Cancer Center and Research Institute, 12902 USF Magnolia Drive, Tampa, FL 33612 USA; 2 Department of Pathology, H. Lee Moffitt Cancer Center and Research Institute, Tampa, FL USA; 3 Department of Chemical Biology, Ernest Mario School of Pharmacy, Rutgers University, Piscataway, NJ USA

Retraction of: *Scientific Reports* 10.1038/s41598-020-80911-z, published online 26 January 2021

Editors have retracted this Article.

An investigation at the Moffitt Cancer Center confirmed that Figures 2a and 6f of this Article contain multiple instances of overlaps representing different experimental conditions. Original data for Figure 2a was no longer available; the original data for Figure 6f was available and corroborated the concerns about the figure content.

Domenico Coppola, Chung S. Yang, and Mokenge P. Malafa did not respond to the correspondence from Editors about the retraction. The Editors were not able to confirm the contact details for Kazim Husain.

